# Effects of exergames on mood and cognition in healthy older adults: A randomized pilot study

**DOI:** 10.3389/fpsyg.2022.1018601

**Published:** 2022-11-07

**Authors:** Beatrice Moret, Massimo Nucci, Gianluca Campana

**Affiliations:** ^1^Department of General Psychology, University of Padova, Padova, Italy; ^2^Human Inspired Technology Research Centre, University of Padova, Padova, Italy

**Keywords:** exergame, cognitive-motor training, cognitive improvement, innovative training, healthy aging

## Abstract

The elderly population is increasing and the implementation of stimulating training to promote active aging has become a research issue. This study aimed at investigating the effects of a cognitive-motor exergame training on cognitive functions and mood, in healthy older adults. A randomized controlled pilot study was conducted to compare a cognitive-motor exergame training with a passive control group. The training consisted of 8 sessions of 45 min each, including 10 interactive activities focused on several cognitive functions such as memory, processing speed and executive functions, all requiring motor planning and execution. A total of 57 participants were administered a battery of cognitive tests before and after the training. A mixed-effect ANOVA with group (experimental vs. control) as between factor and time (pre-and post-test) as within factor, was performed to evaluate the effect of the exergame training on cognitive abilities and mood. Results showed significant interaction effects in processing speed [*STROOPC: F* (_1,53.4_) = 9.04, *p* = 0.004, *R*^2^ = 0.82], inhibition [*3backs’ false alarms: F* (_1,47.5_) = 5.5, *p* = 0.02, *R*^2^ = 0.79], and mood [*Beck Depression Inventory: F* (_1,55_) = 4.15, *p* = 0.04, *R*^2^ = 0.6]. Even though post-hoc analyses did not provide statistical evidence supporting the interactions, overall data showed a trend toward better scores only for the experimental group, suggesting a potential improvement in information processing speed, working memory and mood. Exergaming may be a motivating and enjoyable approach to healthy and active aging.

## Introduction

In recent years the percentage of the elderly population has increased and it continues to grow (World Health Organization, 2015). As a result of the aging process, the physiological decline in motor and cognitive functions inevitably leads to a reduction of daily life functioning, which causes disability and, most likely, costs in terms of independence (Anton et al., 2015), with further implication for the health care system (Harada et al., 2013). This decline is a consequence of brain structural changes consisting of cortical thinning and functional connectivity reduction (Fjell et al., 2009; Pfefferbaum et al., 2013), specifically in the frontal areas, crucial for higher-order cognitive functions such as information processing speed, and executive functions.

Therefore, promoting healthy aging has become necessary. Several studies have shown that the aging brain remains plastic and elderly performance can be improved by practicing systematic motor or cognitive training (Kramer et al., 1999; Shubert et al., 2010). A comprehensive review showed several environmental factors implicated in the reduction of age-related cognitive decline including cognitive and physical fitness training (Kramer et al., 2004).

An efficient way to maintain healthy life lies in the ability to continue to practice physical and cognitive tasks. The combination of physical and cognitive training is often mentioned as dual-task training (Schaefer and Schumacher, 2011; Wollesen and Voelcker-Rehage, 2014). Dual tasking involves the combination of two motor tasks with different goals, or a motor task with a cognitive task (Broeder et al., 2014), such as exergaming. It has been shown that simultaneous or dual-task cognitive and motor training, like in real-life situations, may offer greater cognitive benefits (Law et al., 2014; Levin et al., 2017; Netz, 2019) than performing them separately (Tait et al., 2017). This may be due to numerous reasons such as the similarity of physical-cognitive training with most of the daily life activities; in fact, physical activity seems to encourage neuroplasticity (Kempermann et al., 2010; Hötting and Röder, 2013; Budde et al., 2016); also, combining physical activity with cognitive training could be crucial in driving and consolidating the potential synergistic effect (Gheysen et al., 2018). Moreover, the cognitive-motor intervention might support the growth of cognitive reserve, the ability to make flexible and efficient use of cognitive networks when performing tasks (Stern, 2002), by increasing the efficiency of the cognitive networks underlying executive control (Stern, 2012). Furthermore, people with a higher cognitive reserve can cope with neuronal damage better than people with lower cognitive reserve matched for level of pathology and brain reserve (Steffener and Stern, 2012). These findings have led to the creation of several types of interventions to slow down the process of cognitive decline and, where possible, to the improvement of cognitive functions and quality of life.

A novel promising behavioral method to promote healthy aging is the so-called exergame. The exergames are a type of videogames that combines the movements of the body with the activity of the game through visual and graphic support that allows to interact in the context of the game and to receive feedback (Oh and Yang, 2010). The exergames include physical exercise tasks and they require cognitive abilities to be executed, this makes them an optimal solution to perform motor and cognitive training simultaneously (Barcelos et al., 2015; Eggenberger et al., 2015, 2016; Gavelin et al., 2021). Exergaming has been shown to offer many advantages when practiced by healthy older adults. It can motivate exercising more, it promotes social interactions (Brox et al., 2011) and also, it has been demonstrated to improve many domains: cognitive functions (Stojan and Voelcker-Rehage, 2019; Gallou-Guyot et al., 2020; Raichlen et al., 2020), mostly executive functions (Wollesen et al., 2020), and spatial navigation (Oliveira et al., 2021); physical functions (Street et al., 2017; Kappen et al., 2019), such as step execution (Pichierri et al., 2012); and, in addition to cognitive and physical functions individually, it also improves dual-task execution (Gallou-Guyot et al., 2020).

Studies compared exergaming to canonical cognitive or physical training, showing controversial results especially due to the different types of activity requested. A recent review (Stojan and Voelcker-Rehage, 2019) showed that exergame training effects appear the same or moderately more effective than other physical practice on cognitive enhancement in healthy older adults.

In any case, to understand the effects of exergaming, it is fundamental to contextualize the mode of exercises involved. To do so, we will refer to the model structure proposed by Netz (2019), illustrating the “modes” of exercise related to cognition and the different underlying components involved. Firstly, the exercise modes could be divided into two categories based on the inherent type of energy demanded to carry out the activity (Voelcker-Rehage and Niemann, 2013): physical training (metabolic demanding) vs. motor training (neuromuscular demanding). The physical training activities (aerobic and strength exercises) are normally repetitive and automatic and require high metabolic energy and relatively low neuromuscular effort. On the other hand, motor training (balance, coordination, and flexibility) involves higher neuromuscular demand and relatively lower metabolic demands. Moreover, in the latter, higher-level cognitive processes are needed (Netz, 2019). This theoretical classification is supported by animal studies (Kleim et al., 1998) and neuroimaging human studies (Voelcker-Rehage and Niemann, 2013) that showed physical and motor fitness might affect not only different cortical areas but also competing cortical networks, reflecting different relations to the cognitive outcomes.

Exergaming might include motor or physical training, or both combined with cognitive training, in other words, a combination of physical and cognitive activities, also known as dual-task.

We chose a cognitive-motor exergame with the purpose of inducing a synergistic effect (Gheysen et al., 2018) supported by the possibility to intervene even later in life in order to enrich the cognitive reserve, slow age-related physiological cognitive decline, and promote healthy aging (Stern, 2013). In fact, the cognitive reserve seems to facilitate cognitive performance even in brain impairment acting as a dynamic and adaptable mechanism in the brain (Mondini et al., 2016).

Therefore, this study aimed at maintaining and possibly enhancing cognitive flexibility and attitude. To do so, we investigated if playing cognitive-motor exergame could lead to cognitive and mood changes in healthy older adults. We hypothesized that the participants who performed the exergame training would improve in cognition, specifically in executive functions, due to the activities trained, compared to participants living their daily routine life (with no training). Moreover, since this exergame is an interactive motivating game, we expected an improvement in mood compared with participants not performing it.

This is the first exploratory study which proposes an experimental design using a commercial exergame that trains cognitive functions. Since it is an innovative study, a specific user experience questionnaire investigating exergame training experience has been created based on the System Usability Scale (SUS; Brooke, 1996).

## Materials and methods

### Participants

For the effect size, *a priori* power analysis was performed using the software program G * Power 3.0.10 (Faul et al., 2009). Given an effect size Cohen’s *f* of 0.25 corresponding to Cohen’s *d* = 0.5, an intermediate effect size according to Cohen’s (Cohen, 1988) criteria, *α* of 0.05 (one-sided) and a power of 85%, a sample size of *n* = 38 (a minimum of 19 per group) was determined. However, taking into consideration a certain number of participants not meeting the inclusion criteria or dropping out, a larger sample size was taken into consideration. A total of 70 older adults interested in taking part in the study spontaneously contacted the principal investigator after reading an informative promoted at the “Ufficio Attività Creative per la terza età” (Office of Creative Activities for the Third Age of the Municipality of Padova). The candidates were first informed about the procedure of the research and then inclusion criteria were assessed: (a) age 65 or above; (b) no history of neurological, psychiatric or addictive disorders; (c) normal or corrected-to-normal hearing and vision; (d) absence of cognitive decline [Mini Mental State Examination (MMSE) ≥ 24]; (e) no severe depressive symptoms (BDI-II > 30); the last two criteria were assessed the first meeting in person; in case of a BDI score > 10, the assessing psychologist made sure that the symptoms did not cause clinically significant distress or impairment in social, occupational, or other important areas of functioning, which is basic criterion of DSM 5 for making diagnoses. Cognitive reserve was assessed with the Cognitive Reserve Index (CRI) questionnaire (Nucci et al., 2013).

Eight persons did not meet the inclusion criteria, so the sample size was reduced to 62. A 2:1 randomization procedure in favor of the experimental group was applied for a number of reasons including ethical reasons (maximizing participants’ exposure to the treatment), the expectation of a higher number of drop-out in the more demanding and time consuming exergame training condition, increasing the power of post-hoc tests (Dumville et al., 2006). As a result of this procedure, we allocated 40 participants to the exergame training group and 22 participants to the control group.

Two persons dropped out during the training sessions for personal reasons, and 3 lacked the final assessment. Of the remaining 57 participants who completed the study, 38 had been assigned to the exergame training and 19 to the control group.

Participants were informed about their right to withdraw from the study at any time.

Participants’ characteristics, divided by group, are presented in Table 1.

**Table 1:** Participants’ demographic and baseline characteristics.

Group	Exergame training	Control	Test, value of *p*
*N*	38	19	
Gender			
Women	29 (76.3%)	13 (68.4%)	*X*(1) = 0.45, value of *p* = 0.49
Men	9 (23.7%)	6 (31.6%)	
Age (years)	70.13 (3.73)	71.11 (3.72)	*t* (112) = 1.31, value of *p* = 0.19
	Range: 65–77	Range: 65–77	
Education			
Primary school	6 (15%)	2 (10.5%)	*X*(2) = 1.9, value of *p* = 0.38
High school	11 (28.9%)	9 (47.3%)	
University degree or higher	21 (55.2%)	8 (42.1%)	
CRI	129.1 (14.3)	128.2 (11.6)	*t* (112) = 0.34, value of *p* = 0.73
MMSE	26.84 (1.07)	26.97 (0.67)	

CRI, Cognitive reserve index; MMSE, Mini mental state examination; mean and standard deviation are reported for age, CRI, and MMSE corrected scores.

The study was approved by the Ethics Committee for the Psychological Research of the University of Padova with protocol n° 2351.

### Procedure

The study took place in a laboratory of the Department of General Psychology, University of Padova. Participants, after being elected, were included and randomized in two groups, all submitted to a baseline neuropsychological evaluation and questionnaires. One group was trained with several activities of the exergaming “Dr. Kawashima’s Body and Brain Exercises”; the second, the passive control group, continued the daily routine, and performed the assessment pre- (T0) and post-evaluation (T1) with the same time interval of the other group; participants in the control group were given the possibility to perform the training thereafter the second assessment (seven participants decided to perform the training; the data were not included in the dataset). The total protocol will last 2–3 weeks, 3–4 sessions per week for a total of 10 sessions, composed of eight training sessions, plus two sessions of assessment (*T*0 and *T*1). The training will be carried out in sessions of 45 min each. The same psychologist will conduct evaluations at *T*0 and *T*1 and he/she was blinded to the group assignment of each participant; a different psychologist administered the training. A certificate of participation (Appendix A) in recognition of the evaluation and training conducted was given at the end of the evaluation.

All participants gave written informed consent according to the Declaration of Helsinki.

### Assessment

The first-day assessment includes the initial welcome, the explanation of the study research providing the oral and written informed consents, and an initial screening assessment of the elderly person (see including and excluding criteria) including general cognitive abilities evaluation using the MMSE (Magni et al., 1996), considering a score (corrected for age and education) ≥ 24 for being eligible for the study. MMSE is one of the most often used brief screening tools for providing an overall measure of cognitive impairment often setting <24 as cut-off to select patients with suspected cognitive impairment or dementia (Tombaugh and McIntyre, 1992). Thus, a comprehensive neuropsychological battery and self-report questionnaires were administered to all participants at the baseline and after the training. The available parallel forms of the tests were counterbalanced among participants.

The whole meeting lasted from 70 to 90 min.

Cognitive performance was assessed with “paper and pencil” and computerized tasks investigating different cognitive domains. Tests and self-report questionnaires are explained in detail and assembled for cognitive functions investigated:

#### Working and spatial memory

*Digit Span*, in two subtests: *Forward Digit span (DS-F)* evaluates the functioning of the working memory and *Backwards Digit span (DS-B)* requires a short-term verbal memory span and the ability to manipulate and update verbal information while in temporary storage (Orsini et al., 1987).

*N-back Test*, specifically *2-back and 3-back* working memory tasks using letters (Postle et al., 2000; Jaeggi et al., 2010); temporary storage of the material and manipulation of the information are involved;

*Spatial Recall Test (SPART)* (Rao, 1990) assesses visual memory acquisition through a 6 × 6 checkerboard with 10 checkers randomly placed which the participant has to replace after 10 s of presentation. The test has a parallel form. The task is repeated after 30 min in a delay recall trial (SPART-D).

#### Attention, executive functions, processing speed, and inhibition

*Stroop Color–Word test* (Brugnolo et al., 2016) consisted of three tables with a parallel form (Word items, Stroop-W, Color items, Stroop-C, and Color Word items, Stroop-CW); the participant is given 30 s for each table to read or express the maximum items as possible; sustained attention and some aspects of executive functions, such as the ability to elaborate relevant and irrelevant dimensions in parallel and to inhibit an automatic response (Stroop-CW) are evaluated;

*Symbol Digit Modalities test* (SDMT; Nocentini et al., 2006): according to the oral version administered the participant is given 90 s to verbalize as many symbol-number associations as possible to evaluate sustained attention and processing speed of information;

*Trail Making Test (TMT-A and TMT-B*; Amodio et al., 2002) provides information on visual search, scanning, processing speed, mental flexibility and attention shifting.

#### Psychomotor speed and motor control

*Simple Reaction Time* (SRT; a single stimulus) and *Go-NoGo* task, both created with Psychotoolbox in Matlab, consisting of 40 consecutive trials to measure the reaction times and 50 trials, 40 Go trials (80%) and 10 NoGo trials (20%), to investigate inhibition.

#### Questionnaires (self-perceived mood and cognitive efficacy)

*Beck Depression Inventory II* (BDI-II; Sica and Ghisi, 2007) consists of 21 items relating to depressive symptoms such as hopelessness, irritability and cognitive symptoms;

*Cognitive Failure Questionnaire* (CFQ; Broadbent et al., 1982). It consists of 25 questions regarding small errors and absent-mindedness in everyday lives. It is used to evaluate the effectiveness of the intervention on participants’ daily functioning.

The tests and questionnaires above mentioned were administered in the following order: DS-F, DS-B, TMT-A, TMT-B, SPART, SDMT, 2-back, 3back, SRT, Go-NoGo, SPART-D, STROOP, BDI-II, CFQ. The order of the tests was maintained fixed for two reasons: firstly, in the battery, a version delay test was administered (START-D), so the administration interval time should remain similar among the two assessments; secondly, we would like the participants to be affected by equal attentional effort during the all tests administration in both the two assessments.

#### User experience questionnaire

The 14 items self-report questionnaire based on a Likert scale (from 0 equal to strongly disagree to 4 equal to strongly agree) has been created specifically for this exploratory study in order to investigate the utility and effectiveness of the cognitive exergame and system devices used. The questionnaire takes its cue from the SUS (Brooke, 1996), a widely used questionnaire to evaluate a user’s experience and usability with a new system. Since usability has not univocal possible meaning, it follows that its definition may be dependent on the way in which usability is defined in terms of specific measures. In this context, we identify three mainly participants’ outcome measures: in the first place, usability features should encompass effectiveness, the competence in completing the activity using the exergame, secondly, the efficiency which refers to the level of resources necessary to exergaming and lastly, satisfaction, general users’ gratification (Brooke, 1996). Moreover, at the end of the questionnaire, there were two open questions: one investigated any possible expectations met or not, and the latter, gave the participants the possibility to add a comment regarding their experience exergaming (See Appendix B).

### Exergame training

The training took place in a laboratory furnished with a TV screen 40″ liquid-crystal display (Samsung UE40K5510AKXZT) with a 1920 × 1980 resolution, an Xbox-360 console and a Kinect device. Each participant, standing approximately two meters away from the TV screen, performed the training individually under the supervision of a trained research assistant. The Kinect sensors provide participants with real-time audio-visual feedback.

The first training session was dedicated to the habituation phase with the exergaming modality and the Xbox equipment. Fruit Ninja exergame was selected for this first familiarization process, choosing three different activities: Zen, Classic, and Arcade. The games were repeated several times with increasing difficulty levels until the player feel confident in playing. The purpose of this first game session was for the participants to manage how to act in response to the exergame system using and to familiarize themselves with the virtual environment before beginning to real cognitive-motor training.

The exergame “Dr Kawashima: brain and body exercises” has been selected as a combination of motor training (requiring proprioception and eye-hand/leg coordination) and cognitive training including several cognitive tasks (working memory, attention, executive functions), necessitating the ability to handle visual and spatial information.

Specifically, it consists of interactive video game-based cognitive and physical-related exercises, demanding full-motion capabilities. Each activity trains several cognitive functions such as memory, reaction times, processing speed and executive functions, besides requiring motor planning and execution. The exergame provides each activity with three difficulty levels and the progression is determined by the player’s performance. To move to the next level a C score is necessary. Players can track their daily results ranging from A (excellent performance) to an F (bad performance). Among the 20 activities the exergame offers, 10 games were selected and kept in an identical order for all sessions of training (see Table 2). The activities order was chosen to be easily selectable.

**Table 2:** Illustration of the 10 exergame activities and the cognitive functions related.

Exergame activity	Cognitive functions
1-Numerical balloons	Visual search, selective attention
2-Coloured balloons	Stroop effect, selective attention, inhibition
3-Traffic policeman	Executive functions, inhibition
4-Turbulent mice	Selective attention, inhibition
5-Turn and discover	Visuospatial working memory
6-Memory step	Spatial working memory
7-Golden ball	Processing speed
8-What time is it	Executive functions, processing speed
9-Radar	Processing speed, working memory
10-Perfect couple	Visual search, processing speed

### Data analyses

All analyses were performed in the R environment. Sociodemographic differences between the two samples differently according to the data using Student’s t-test for continuous variables and the chi-square test for categorical variables were performed. All tests were two-tailed with alpha level *p* < 0.05. Means, standard deviations, counts, and percentages for continuous and categorical variables, respectively were calculated.

To verify changes over time, differences between the trained and control group, and the differential impact of the group over time (group × time), we performed mixed-effect ANOVA model (with Restricted Maximum Likelihood Analysis–REML estimation and na.omit option, i.e., stripping any observations with missing values in any variables). Variables of interest were the tests of the neuropsychological battery described above (e.g., Digit Span, Trial Making Test, Selective Reminding Test, Symbol Digit Modality Test, Stroop test, N-Back, etc.).

Effect sizes (Table 3, see Results section), reported as the conditional (pseudo) *R*^2^ (Nakagawa et al., 2017) to measure model fit, including both fixed and random factors, were calculated. *Post hoc* analyses were conducted using False Discovery Rate adjustment (Benjamini and Hochberg, 1995) of value of p. Statistical analyses were performed in R (R Core Team, 2020) using the “lmerTest” (Kuznetsova et al., 2017) and “MuMIn” packages (Barton et al., 2015). Concerning the user experience questionnaire, a percentage frequency distribution, that specifies the degree of agreement and disagreement in percentage for each item for each of the five levels, was calculated.

**Table 3:** Descriptive and inferential analysis of all measures tested.

Measures	Descriptive				Inference				
	Exergame training		Control						
	*T*0	*T*1	*T*0	*T*1	Variable	df	*F*	value of *p*	Cond.*R*^2^
Memory tasks									
DS-F	5.71 (1.14)	5.89 (0.98)	6.05 (0.91)	6.11 (1.2)	Group	155	0.64	0.426	0.52
					Time	155	1.13	0.292	
					Group*Time	155	0.2	0.657	
DS-B	4.34 (1.12)	4.63 (1.34)	4.68 (0.89)	4.74 (0.99)	Group	155	1.39	0.244	0.6
					Time	155	0.6	0.441	
					Group*Time	155	0.67	0.418	
2back Go	21.5 (2.33)	22.57 (2.84)	20.89 (4.34)	21.95 (2.76)	Group	147.5	8.3	0.006^*^	0.68
					Time	148.8	0.54	0.465	
					Group*Time	147.5	0	0.966	
2back FA	5.03 (4.55)	5.57 (5.11)	7.21 (9.6)	6 (6.23)	Group	146.7	0.34	0.562	0.63
					Time	149.3	0.77	0.383	
					Group*Time	146.7	0.85	0.361	
3back_Go	17.68 (4.36)	18.51 (4.45)	16 (5.22)	17.11 (4.32)	Group	153.6	1.58	0.214	0.34
					Time	154.5	2.03	0.16	
					Group*Time	153.6	0.01	0.909	
3back_FA	6.7 (5.19)	5.59 (3.5)	4.18 (2.38)	6 (5.78)	Group	147.6	0.21	0.649	0.79
					Time	149.9	0.21	0.646	
					Group*Time	147.6	5.5	0.023^*^	
SPART	18.03 (4.76)	18.89 (5.65)	18.05 (3.69)	18.79 (4.94)	Group	155	0.89	0.349	0.26
					Time	155	0	0.972	
					Group*Time	155	0.01	0.939	
SPARTD	6.13 (2.33)	6.45 (2.34)	6.26 (2.38)	5.95 (1.99)	Group	155	0	1	0.44
					Time	155	0.11	0.736	
					Group*Time	155	0.85	0.36	
Attention, executive functions, processing speed and inhibition									
STROOPW	66.45 (8.73)	68.45 (9.45)	63.37 (10.51)	65.58 (10.36)	Group	155	3.46	0.068	0.66
					Time	155	1.49	0.227	
					Group*Time	155	0.01	0.926	
STROOPC	46.32 (6.81)	49.87 (6.68)	44.22 (8.43)	44.58 (9.28)	Group	153.4	5.29	0.025^*^	0.82
					Time	154.3	2.58	0.114	
					Group*Time	153.4	9.04	0.004^*^	
STROOPCW	24.03 (5.43)	26.11 (6.5)	23.61 (6.55)	24.47 (6.7)	Group	154.8	3.98	0.051	0.68
					Time	155.5	0.37	0.543	
					Group*Time	154.8	0.84	0.362	
					Group	155	5.48	0.023^*^	0.8
					Time	155	0.38	0.538	
					Group*Time	155	0.13	0.715	
TMT-A	44.5 (15.4)	37.34 (11.39)	47.26 (18.44)	44.05 (16.76)	Group	155	6.94	0.011^*^	0.59
					Time	155	1.62	0.209	
					Group*Time	155	1.01	0.32	
TMT-B	108.29 (35.36)	99.74 (25.61)	111.11 (47.68)	103.89 (48.63)	Group	155	4.29	0.043^*^	0.74
					Time	155	0.13	0.723	
					Group*Time	155	0.03	0.861	
Psychomotor speed and motor control									
SRT median	0.29 (0.07)	0.28 (0.05)	0.28 (0.05)	0.3 (0.04)	Group	154.7	0.37	0.545	0.32
					Time	155.1	0.35	0.557	
					Group*Time	154.7	2.38	0.129	
Go median	0.38 (0.04)	0.38 (0.04)	0.37 (0.03)	0.39 (0.04)	Group	155	1.71	0.196	0.66
					Time	155	0.13	0.716	
					Group*Time	155	1.88	0.176	
HITs (Go)	35.74 (4.38)	35.97 (4.4)	35.53 (5.15)	35.63 (4.84)	Group	155	0.15	0.702	0.76
					Time	155	0.05	0.821	
					Group*Time	155	0.02	0.883	
FAs (NoGo)	1.18 (1.43)	0.74 (0.79)	1.21 (1.18)	0.89 (0.94)	Group	155	4.25	0.044^*^	0.34
					Time	155	0.13	0.722	
					Group*Time	155	0.13	0.724	
Questionnaires (self-perceived mood and cognitive efficacy)									
BDI	7.16 (5.91)	4.47 (4.69)	7.89 (5.18)	8.05 (5.42)	Group	155	3.28	0.076	0.6
					Time	155	2.66	0.109	
					Group*Time	155	4.15	0.046^*^	
CFQ	33.21 (13.39)	29.05 (12.58)	35.21 (14.21)	35.05 (12.03)	Group	155	2.42	0.125	0.72
					Time	155	1.39	0.244	
					Group*Time	155	2.08	0.155	

Means and standards deviations for all measures are indicated; T0, pre-test; T1, post-test; df, degree of freedom; DS-F, Digit Span Forward; DS-B, Digit Span Backward; SPART, Spatial Recall Test; SPART-D, Spatial Recall Test—Delay; STROOPW, Stroop Word; STROOPC, Stroop Colour; STROOPCW, Stroop Colour-Word; SDMT, Symbol Digit Modalities test; TMT-A and TMT-B, Trail Making Test A and B; SRT, Simple Reaction Time; HITs, correct responses; FAs, False Alarms; BDI-II, Beck Depression Inventory II; CFQ, Cognitive Failure Questionnaire.

* *p* < 0.05. The bold represents a significant Group*Time interaction.

## Results

**Figure 1:**
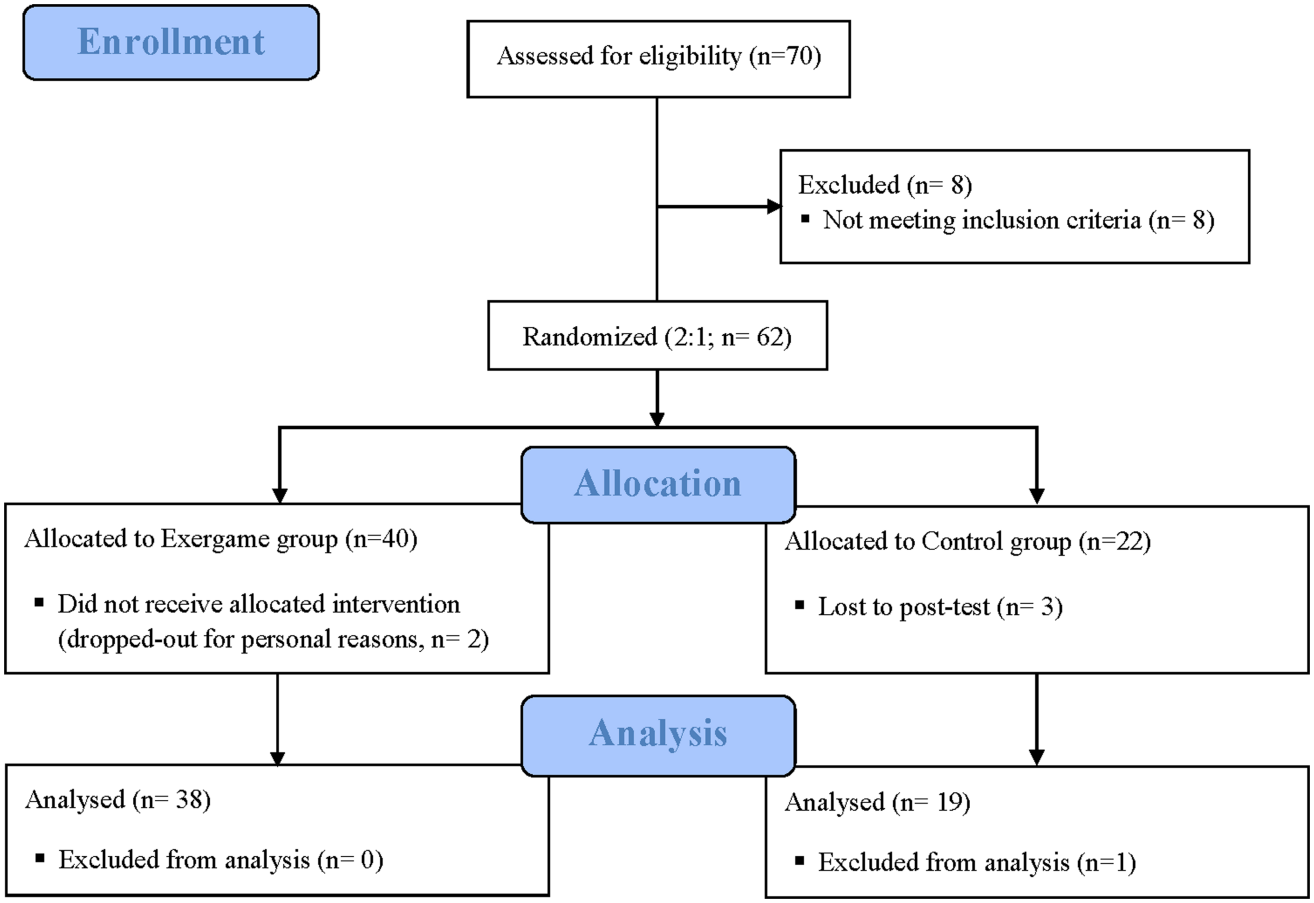
Participants’ flow and study design. Participants were randomly assigned to either experimental and control group. Exergame group was trained over eight intensive sessions for 45 min a day. A comprehensive neuropsychological battery was administered to both groups.

The analyzed data sample was composed of 57 people of whom 38 performed the exergame training and 19 were control group members. Participants’ flow is presented in Figure 1. The intervention and control groups showed no significant sociodemographic differences (see Table 1). In both groups, the percentage of females was higher than males, in the exergame group it corresponded to 76.3% and in the control group to 68.4%. The data collected showed a very high average CRI value of around 130, almost identical in the two groups (probably due to the recruitment modality occurred through advertisements placed in the office of creative activities for the third age of the municipality of Padova, with spontaneous participation).

### Neuropsychological battery and questionnaires

**Figure 2:**
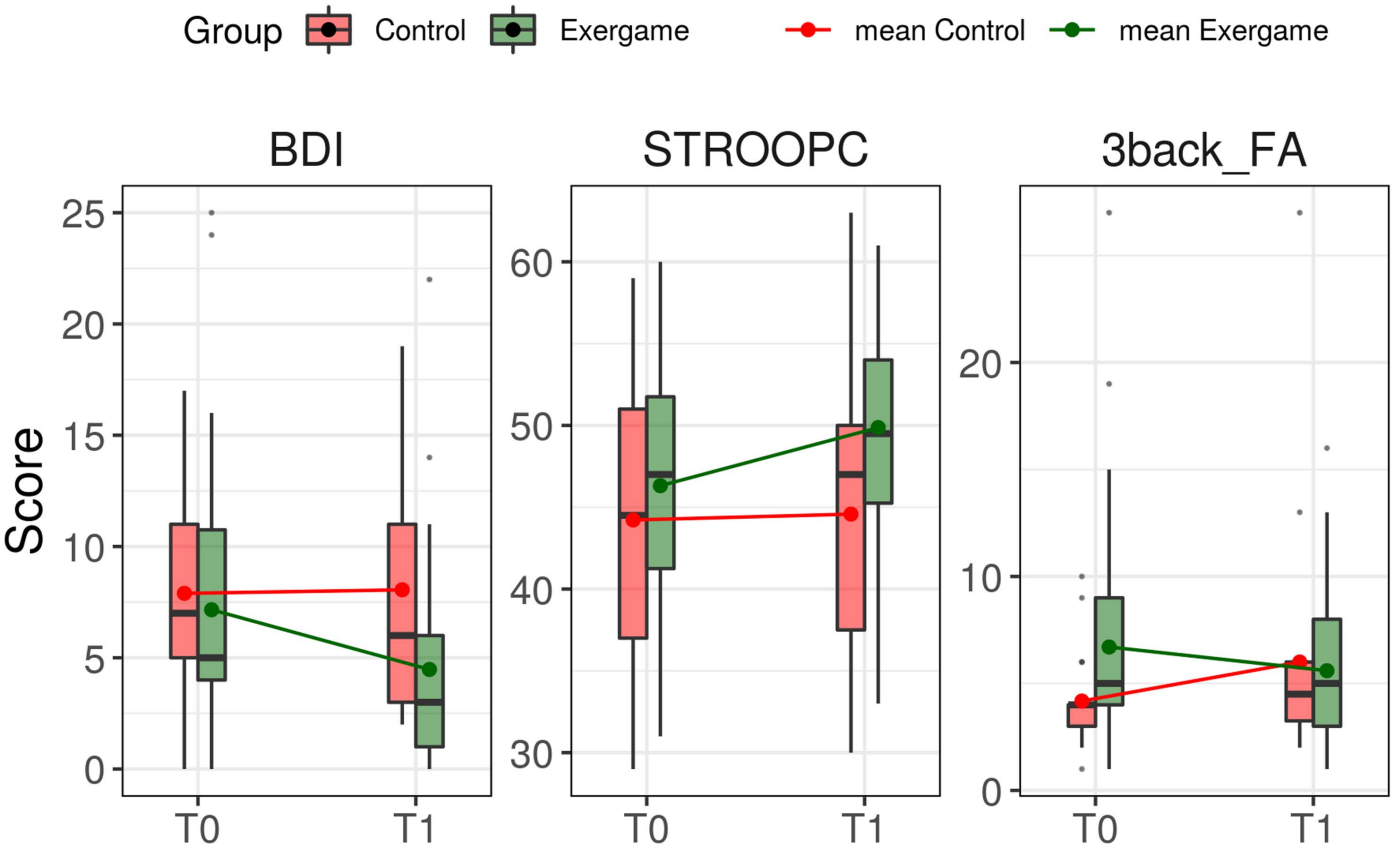
Cognitive outcomes in pre-and post-test assessments. Improvements were shown at T1 in all three tests just for Exergame group. No improvements were found for the Control Group. BDI-II: Beck Depression Inventory II; STROOPC_ Stroop Colour; 3back_FA: 3back_False Alarms.

To evaluate the effect of the exergame training on cognitive abilities, a mixed-effect ANOVA model with Group (training and control participants) and Time (T0 and T1) on all measures was carried out. The results revealed significant main effects (not pertaining to the hypotheses) in several variables. The value of conditional *R*^2^ is both for fixed and random effects (see Table 3). Two subtests and the mood questionnaire showed significant interaction effects in expected directions: *STROOPC F* (_1,53.4_) = 9.04, *p* = 0.004, *R*^2^ = 0.82; *3back_FA F* (_1,47.5_) = 5.5, *p* = 0.02, *R*^2^ = 0.79; and *BDI F* (_1,55_) = 4.15, *p* = 0.04, *R*^2^ = 0.6 (see Table 3; Figure 2).

The differences between T0 and T1, tested separately for the training and control groups through simple t-tests, resulted statistically significant only for the training group for STROOPC *t*(74) = −2.29, *p* = 0.025 and BDI *t*(74) = 2.1929, *p* = 0.031 measures. However, after adjusting for multiple comparisons using the False Discovery Rate method (Benjamini and Hochberg, 1995), significances are not maintained.

### Exergame’s user experience questionnaire

This 14 items self-report questionnaire explored the user’s experience playing the exergame. The results are illustrated in percentage of frequency distribution for each of the Likert scale levels: 0 = Strongly disagree, 1 = Disagree, 2 = Neither agree nor disagree, 3 = Agree, and 4 = Strongly agree (see Appendix B). Interestingly, concerning the usability of the exergame in terms of user-friendliness (items 1, 9, and 10) more than 80% of the participants agreed in it. Also, more than 80% considered the training performed useful (item 4) and even more than 90% believed that this interactive modality made it more motivating (item 6), and it could become an effective way to carry out rehabilitation training (item 12). Regarding participants’ comments on any expectations many promising feedbacks were provided: “I had a great time,” “It seems to me I have improved even though old age plays tricks on me,” “I understand my memory limits better and I am more focused.”

## Discussion

This exploratory study consisted of a 2/3 week intensive training aimed at promoting active healthy aging by boosting cognitive flexibility through an enjoyable motivating exergame. This innovative protocol compared a simultaneous cognitive-motor exergame training with a merely daily routine activity. We expected the participants who completed the exergame training would have performed better at the neuropsychological tests, specifically for executive functions and processing speed (Stojan and Voelcker-Rehage, 2019; Wollesen et al., 2020), also due to the dual task modality of the exergame which required a certain level of cognitive exercise load with rising difficulties (Wollesen and Voelcker-Rehage, 2014; Ogawa et al., 2016). Moreover, an improvement in mood symptoms following the exergame intervention was expected (Drazich et al., 2020). Lastly, this cognitive-motor exergame training was performed for the first time by healthy older adults thus, user experience was investigated through a questionnaire created on purpose, to collect experiences related to effectiveness, usefulness, enjoyment and satisfaction, and any critical issues encountered by the participants.

### Is cognitive exergaming able to boost cognitive performance and mood?

Although this exploratory study did not provide statistical evidence of a beneficial effect of the exergame on cognitive functions and mood, after eight sessions of exergame training, a positive trend in three measures, Stroop Colour, 3back-false alarms, and Beck Depression Inventory, was observed in the expected direction.

With respect to cognitive tasks, there was an increasing trend in Stroop color accuracy (naming as many items-colored squares-as possible in 30 s) suggesting a faster processing of information of displayed stimuli. The exergame training of the current study included the activity “Coloured balloons” consisting of a Stroop game requiring not only to discriminate among a number of stimuli but also to increase the speed of processing them. Such improvement was not found for Stroop Word task (reading as many words-colors-as possible in 30 s) reflecting instead the automation of reading, that has not been trained in any of the exergame activities. Overall, this result could reflect the benefits of the exergame as observed in other studies finding better executive control and processing speed tasks after the training (Maillot et al., 2012; Eggenberger et al., 2015; Schoene et al., 2015).

Moreover, a positive trend was observed just for the 3back task, specifically a reduction of FAs while maintaining the same Hits score. The n-back task is a tool widely used to evaluate working memory (WM). Also, Liao et al. (2021) showed a significant WM improvement in just the exergame training group compared to the combined physical exercise training (Liao et al., 2021).

To accomplish this difficult task, selective attention to both maintain and manipulate information in WM is needed. The FAs reduction, representing an increase in the accuracy in rejecting no-targets, could reflect an executive control processing increase which plays a crucial role in the n-back task (Gajewski et al., 2018), allowing better continuous updating to keep track of the target stimulus (Gazzaniga et al., 2009), likely induced by the engaging exergame training.

Concerning mood, as shown in a systematic review and meta-analysis by Huang et al. (2022), exergame-based exercise training had beneficial effects, for both populations with and without depressive symptoms, with mixed results for the latter. Although the factors involved are not fully known and the results are controversial (Drazich et al., 2020), research regarding the impact of exergames on depressive symptoms in the elderly is increasing. Studies suggested a physiological change due to the exergame training, which may result in beta-endorphins increase (Li et al., 2018b), as well as a positive social contribution in terms of social well-being enhancement, such as reduction of loneliness, increased social connection, and positive attitudes towards others (Li et al., 2018a). In the current study, the trend of reduction in depressive symptoms, is also supported by the participants’ conclusive comments—e.g.: “I had a lot of fun,” “I would like to repeat it. Please contact me again!”—suggested that being active, just playing an exergame might have a beneficial effect on the emotional state of the elderly.

More generally, the results obtained could be likely due to the low power of the tests or the exergame training used was not specific enough to improve the cognitive functions tested. Furthermore, a possible explanation for the lack of overt effects could be due to the low intensity effort required by this exergame training. In this context, as illustrated in the introduction, we classified the exergame chosen as motor training, which required less metabolic demands compared to physical training (Netz, 2019). In this regard, despite exergaming resulted slightly more effective than other physical interventions with positive effects in neurophysiological outcomes in cognitive functions in healthy older adults (Stojan and Voelcker-Rehage, 2019), a moderate physical effort induced by exergaming has been demonstrated necessary to produce biological changes and generate important functional adaptations, thus cognitive enhancement (Monteiro-Junior et al., 2016). In addition, as stated in a recent systematic review and meta-analysis by Gheysen et al. (2018), we selected this exergame with the purpose of a synergistic effect combining the motor with the cognitive components, promoted as a modality for preventing and treating cognitive decline in older adults. However, controversial results regarding the benefits of physical activity for cognitive performance in healthy older adults have been shown (Gallou-Guyot et al., 2020). Besides we lacked objective measurements to quantify the energy consumption, we cannot sustain the hypothesis that participants could have taken advantage of the motor modality training (balance, coordination and flexibility) adopted, [which differs from physical training (metabolic demanding; Netz, 2019)], as stated by Voelcker-Rehage et al. (2010).

Moreover, in association with the type of exergame, it is fundamental to consider at which doses beneficial effects might take place. For instance, several reviews (Lampit et al., 2014; Lauenroth et al., 2016; Ogawa et al., 2016) include studies with a similar dose and provide a comprehensive explanation of the influence of different training features such as length, frequency, duration, intensity and level of task difficulty on cognitive performance in healthy older adults. However, a study consisting of 6 weeks of exergame training was not able to enhance cognitive, motor, and sensory functions in healthy old participants (Ordnung et al., 2017). In this regard, a complete review by Cabral et al. (2019), illustrates that, in the short-term (1 day–16 weeks), only a combined approach (aerobic and resistance exercises) with at least moderate intensity will better contribute to improving various mechanisms such as synaptic neuroplasticity, brain volume and connectivity, neurogenesis, and regulation of trophic factors, all believed to support cognitive improvements.

For these reasons, accordingly to previous results, we supposed that the exergame type adopted (Levin et al., 2017) along with the low dose intervention might have contributed to the inconsistency of the results obtained.

Another possible explanation for the lack of robust cognitive improvement may be due to participants’ absence of cognitive difficulties and their very high, above average, cognitive reserve (CR) which might have influenced the margin of improvement. Mondini et al. (2016) showed that those participants who took more advantage of the training were people with low CR, suggesting that CR can influence the result of a cognitive training program (Mondini et al., 2016).

### Players’ exergame experience

As shown in Appendix B, participants gave overall positive perceptions of user experience playing the exergame in terms of user-friendliness, enjoyment, and satisfaction. All the participants showed a willingness to learn how to use it to progress and improve their performance while having fun. Besides this encouraging feedback, some negative aspects concerning the usability system, including perceiving limits have been reported. Understanding the barriers and facilitators in the use of exergames by older adults can guide the adoption of these challenging tools to offer opportunities to increase physical activity (Barg-Walkow et al., 2017) and to design rehabilitative training as shown in recent several studies (Pirovano et al., 2016; Skjæret et al., 2016; Tobaigy et al., 2018; Reis et al., 2019).

### Limitations of the study

Due to the combined nature of the exergame, the assessment should be expanded to investigate the objective effort made, like with an accelerometer and heart rate recording, to quantify the energy consumption, and the subjective perceived exertion, using a self-report questionnaire for better interpreting the results. Also, in line with the literature results, a longer intervention should be implemented. Lastly, some methodological limitations of this study have to be considered such as the lack of an active control group and follow-up measurements to assess the duration of the training effects.

To conclude, with this exploratory study aimed at promoting cognitive flexibility and attitude, positive responses have been collected. The innovative training notions of simultaneous cognitive-motor exergame tended to be enjoyed more by the elderly than traditional training and might lead to an improvement in cognition and mood. Consequently, we recommend simultaneous cognitive exergame training in older adults. The discussion of the efficacy of the exergames should not be limited to testing cognitive abilities, mood and user experience. The ultimate aim is to find methods to improve the autonomy, functionality, and quality of life of the elderly. Further studies should explore the impact of this innovative technology, also comparing it with tailored training and conventional exercises, using a robust methodology in order to improve the quality of the evidence and provide clear and reliable guidelines.

## Data availability statement

The raw data supporting the conclusions of this article will be made available by the authors, without undue reservation.

## Ethics statement

The studies involving human participants were reviewed and approved by Ethics Committee for the Psychological Research of the University of Padova with protocol n° 2,351. The patients/participants provided their written informed consent to participate in this study.

## Author contributions

BM: study preparation and conception, participants’ recruitment, training instruction, data acquisition, statistical analysis, data interpretation, drafting manuscript, and revising manuscript. MN: statistical analysis, data interpretation, and revising manuscript. GC: study preparation supervision, data interpretation, and revising manuscript. All authors contributed to the article and approved the submitted version.

## Funding

BM was supported by a grant from MIUR (Dipartimenti di Eccellenza DM 11/05/2017 n. 262) to the Department of General Psychology, University of Padova-CUP: C96C18000450005. This work was carried out within the scope of the project “Use-inspired basic research,” for which the Department of General Psychology of the University of Padova has been recognized as “Dipartimento di Eccellenza” by the Ministry of University and Research.

## Conflict of interest

The authors declare that the research was conducted in the absence of any commercial or financial relationships that could be construed as a potential conflict of interest.

## Publisher’s note

All claims expressed in this article are solely those of the authors and do not necessarily represent those of their affiliated organizations, or those of the publisher, the editors and the reviewers. Any product that may be evaluated in this article, or claim that may be made by its manufacturer, is not guaranteed or endorsed by the publisher.

## Appendix A

Original Italian version of the certificate of attendance provided to the participants.

## Appendix B

User experience questionnaire (English translated version).
