# Supplementary material for: Effects of exergames on mood and cognition in healthy older adults: A randomized pilot study
Source: Front Psychol. 2022 Nov 7;13:1018601. doi: 10.3389/fpsyg.2022.1018601 (PMC9676977; doi:10.3389/fpsyg.2022.1018601)
Supplement: Supplementary file 1 [file Table_1.DOCX]

| Items | **Strongly disagree** | **Disagree** | **Neither agree nor disagree** | **Agree** | **Strongly disagree** | **N tot (38)** |
| --- | --- | --- | --- | --- | --- | --- |
| 1) I think most people like me could learn to use this exergame easily | 0,0% | 0,0% | 13,2% | 55,3% | 31,6% | 100% |
| 2) I felt comfortable using the exergame | 0,0% | 5,3% | 5,3% | 44,7% | 44,7% | 100% |
| 3) I had to learn many things before I could use the exergame | 26,3% | 21,1% | 23,7% | 23,7% | 5,3% | 100% |
| 4) I think this exergame was useful to me | 0,0% | 2,6% | 15,8% | 36,8% | 44,7% | 100% |
| 5) I think doing exercises with exergame makes my training less heavy | 2,6% | 0,0% | 10,5% | 36,8% | 50,0% | 100% |
| 6) I think that doing exercises with an exergame makes this training motivating | 0,0% | 0,0% | 7,9% | 34,2% | 57,9% | 100% |
| 7) I would be interested in using this exergame outside of this research | 0,0% | 10,5% | 7,9% | 42,1% | 39,5% | 100% |
| 8) If I had it available, I think I would like to use this exergame frequently | 2,6% | 10,5% | 10,5% | 52,6% | 23,7% | 100% |
| 9) It was easy to understand the instructions of the exergame | 0,0% | 0,0% | 15,8% | 36,8% | 47,4% | 100% |
| 10) I found the exergame simple to use | 0,0% | 2,6% | 18,4% | 36,8% | 42,1% | 100% |
| 11) I think that, outside this research lab, I would be able to use the exergame on my own | 5,3% | 0,0% | 18,4% | 39,5% | 36,8% | 100% |
| 12) I think exergame is an effective way to carry out rehabilitation training | 0,0% | 0,0% | 15,8% | 42,1% | 42,1% | 100% |
| 13) Often the kinect did not respond well to my commands | 10,5% | 21,1% | 21,1% | 31,6% | 15,8% | 100% |
| 14) When the exergame did not respond to commands, this was due to the way I gave them | 5,3% | 10,5% | 31,6% | 28,9% | 23,7% | 100% |
