# Supplementary figures and images for: Effects of exergames on mood and cognition in healthy older adults: A randomized pilot study
Source: Front Psychol. 2022 Nov 7;13:1018601. doi: 10.3389/fpsyg.2022.1018601 (PMC9676977; doi:10.3389/fpsyg.2022.1018601)

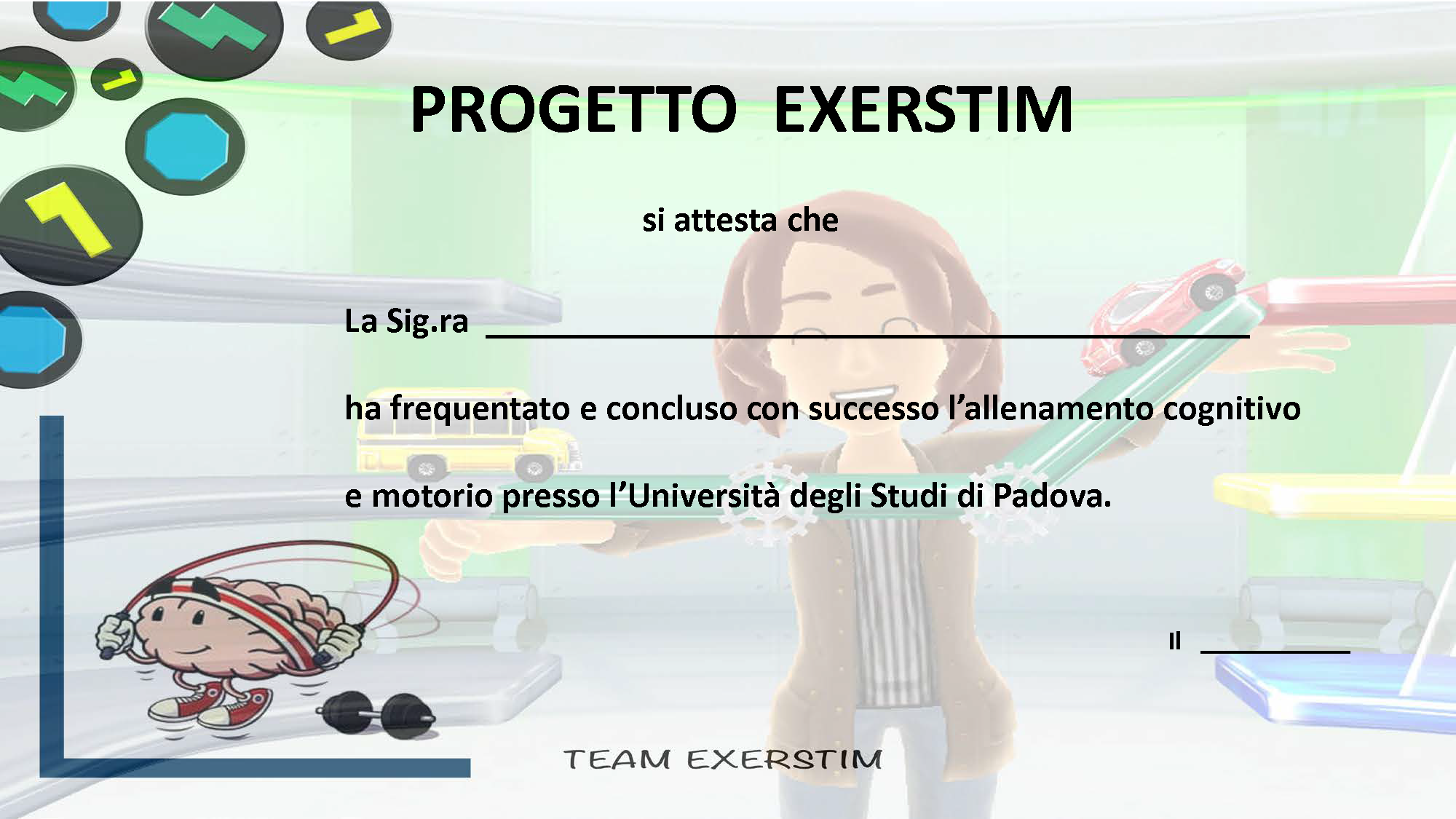

Supplement: Supplementary file 2 [file Image_1.tif]
